# Novel Founder Mutation in *FANCA* Gene (c.3446_3449dupCCCT) Among Romani Patients from the Balkan Region

**DOI:** 10.4274/balkanmedj.2017.0618

**Published:** 2018-01-20

**Authors:** Marija Dimishkovska, Vjosa Mulliqi Kotori, Zoran Gucev, Svetlana Kocheva, Momir Polenakovic, Dijana Plaseska-Karanfilska

**Affiliations:** 1 Research Centre for Genetic Engineering and Biotechnology “Georgi D. Efremov”, Macedonian Academy of Science and Arts, Skopje, Macedonia; 2 Department of Endocrinology, Pediatric Clinic, University Clinical Center, Prishtina, Republic of Kosovo; 3 Department of Pediatrics, St. Cyril and Methodius University, Skopje, Macedonia

**Keywords:** Fanconi anemia, mutation, Balkan region

## Abstract

**Background:**

Fanconi anemia is a rare autosomal recessive or X-linked disorder characterised by clinical and genetic heterogeneity. Most fanconi anemia patients harbour homozygous or double heterozygous mutations in the *FANCA* (60-65%), *FANCC* (10-15%), *FANCG* (~10%) or *FANCD2* (3-6%) genes. We have already reported the *FANCA* variant c.190–256_283+1680del2040dupC as a founder mutation among Macedonian fanconi anemia patients of Gypsy-like ethnic origin. Here, we present a novel *FANCA* mutation in two patients from Macedonia and Kosovo.

**Case Report:**

The novel *FANCA* mutation c.3446_3449dupCCCT was identified in two fanconi anemia patients with Romany ethnicity; a 2-year-old girl from Macedonia who is a compound heterozygote for a previously reported *FANCA* c.190-256_283+1680del2040dupC and the novel mutation and a 10-year-old girl from Kosovo who is a homozygote for the novel *FANCA* c.3446_3449dupCCCT mutation. The novel mutation is located in exon 35 in the FAAP20-binding domain which plays a crucial role in the *FANCA*-FAAP20 interaction and is required for integrity of the fanconi anemia pathway.

**Conclusion:**

The finding of the *FANCA* c.3446_3449dupCCCT mutation in two unrelated FA patients with Romani ethnicity from Macedonia and Kosovo suggests it is a founder mutation in the Romani population living in the Balkan region.

Fanconi anemia (FA) is a rare autosomal recessive or X-linked disorder which is the most frequent cause of inherited bone marrow failure (BMF) along with aplastic anemia (1). Despite the life-threatening BMF and haematological tumours, FA is characterised by a predisposition to solid tumours and congenital anomalies that include: abnormal skin pigmentation; malformations of the thumbs (smaller, duplicated or absent) and forearms; genital organ abnormality; skeletal anomalies; malformations of eyes, ears, heart, digestive system, central nervous system and of the kidneys and urinary tract. Growth hormone deficiency, hypothyroidism and abnormal glucose/insulin metabolism are also common and lead to short stature, obesity and dyslipidaemia (2).

Most FA patients harbour homozygous or double heterozygous mutations in the *FANCA* (60-65%), *FANCC* (10-15%), *FANCG* (~10%) or *FANCD2 *(3-6%) genes, while the minority of FA patients are characterised by mutations distributed in the remaining 12 *FANC* genes (3). Patients with loss-of-function mutations in *FANCA* gene and patients with *FANCG* mutations are considered high risk groups, with more severe or earlier presentation of the disease (4). However, there appears to be no clear relation between the type of the mutation and the phenotype; thus, the mutation type has little prognostic value in *FANCA* patients (5). Most FA patients carry private mutations, resulting in a large heterogeneous list of *FANCA* mutations, including large deletions, small indels, and nonsense, splicing, frameshift and missense mutations, reported in the databases. Furthermore, mutations that recur in one ethnic population have also been described. Recently, we have shown that *FANCA* c.190–256_283+1680del2040dupC is a founder mutation in Macedonian FA patients of Gypsy-like ethnic origin (6).

Here, we report a novel truncating mutation c.3446_3449dupCCCT (p.Met1151ProfsTer65) in exon 35 of the *FANCA* gene identified in two unrelated FA patients with Romani ethnicity from Macedonia and Kosovo.

## CASE PRESENTATION

Patient 1 was a 2-year-old girl from Macedonia, with Romani ethnicity, born small for gestational age, with early presentation of anemia (2.4 years), elevated foetal haemoglobin level (7.1%) and no skeletal or renal anomalies. She had normochromic anemia, thrombocytopenia and pale skin with abnormal pigmentation (café-au-lait). Bone marrow biopsy revealed hypocellular marrow with loss of megakaryocytes and megakaryoblasts. The patient died shortly after the clinical diagnosis from intracranial haemorrhage as a complication of severe thrombocytopenia.

Patient 2 was a 10-year-old girl from Kosovo, with Romani ethnicity, born small for gestational age with an early presentation of megaloblastic anemia and unilateral renal agenesis. At 7 years, she had the bone age expected at 2 years and severe growth hormone deficiency. At this time, treatment with growth hormone, vitamin C and folan was started. After one year of hormone treatment, she gained 9 cm in height and 3 kg in weight. Because of severe anemia and thrombocytopenia, blood transfusions were made.

Genomic DNA was obtained from peripheral blood of the probands and their parents. Due to a suspicion of FA, the patients were screened for the known Macedonian founder mutation, c.190–256_283+1680del2040dupC (exon 3 deletion) in the *FANCA* gene, as already described (6). This mutation was only detected in patient 1 and her mother in a heterozygous state.

Since the clinical presentation of the probands was not explained by this result, targeted resequencing on a MiSeq desktop sequencer was performed using an IlluminaTruSight One sequencing panel (Illumina, San Diego, CA, USA). Libraries were prepared according to the Illumina protocol and data analysis was done as previously reported (7). Variants with a global frequency under 1% according to the 1000 genomes database (www.1000genomes.org/) and the ExAc database (exac.broadinstitute.org/) were also thoroughly investigated, especially truncating variants in the genes associated with anemia and short stature. Targeted resequencing revealed a novel truncating mutation, c.3446_3449dupCCCT (p.Met1151ProfsTer65), in exon 35 of the *FANCA* gene (NM_000135.2), in the heterozygous form in patient 1 and homozygous form in patient 2.

**Figure 1:**
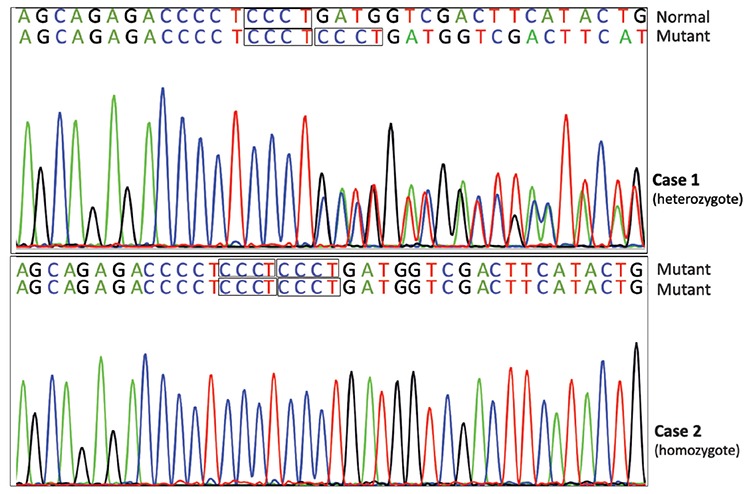
Confirmation of the c.3446_3449dupCCCT mutation in exon 35 in *FANCA* gene in the probands by Sanger sequencing.

The results of the NGS (next generation sequencing) analysis were confirmed by direct DNA sequencing of exon 35 of the *FANCA* gene (Figure 1). PCR and sequencing conditions are available upon request. Direct sequencing showed that the father of patient 1, and both parents of patient 2 were carriers of the *FANCA* c.3446_3449dupCCCT mutation. The genotypes of the probands and their parents are shown in Table 1.

Informed written consent was obtained for all involved patients and the study was approved by the ethics committee.

## DISCUSSION

FA is a rare inherited genetic disorder with an incidence of 1-5 per million and estimated carrier frequency of 1 in 300 individuals (8). In Macedonia, only three patients (from Gypsy-like ethnic population) were diagnosed with FA and all were homozygotes for the exon 3 deletion (c.190–256_283+1680del2040dupC) of the *FANCA* gene.

The two patients described here had a clinical presentation of FA, but only one was heterozygous for the exon 3 deletion of the *FANCA* gene. Targeted resequencing on DNA from both revealed a novel truncating mutation c.3446_3449dupCCCT (p.Met1151ProfsTer65) in exon 35 of the *FANCA* gene (in heterozygous state in patient 1 and in homozygous state in patient 2).

The c.3446_3449dupCCCT mutation shifts the reading frame and causes termination of the protein 65 codons downstream, resulting in a protein that is 241 amino acids shorter than the wild type. According to the Atlas of Genetics and Cytogenetics in Oncology and Haematology database, the mutation is located in the N-terminal, FAAP20-binding, domain (www.atlasgeneticsoncology.org/). This domain (residues 1095 to 1200) plays a crucial role in the *FANCA*-FAAP20 interaction, which is required for the stability of both proteins and for integrity of the FA pathway. This interaction is also involved in *FANCD2 *monoubiquitination (9,10). We assume that this mutation causes a loss of protein function due to premature truncation and abolishment of the *FANCA*-FAAP20 interaction.

The c.3446_3449dupCCCT mutation has not been described in the scientific literature or in electronic databases (ClinVar, LOVD, ExAC). Its finding in our two unrelated FA patients from Macedonia and Kosovo suggests that it is a new founder mutation among the Romani population living in the Balkan region.

## Figures and Tables

**Table 1:**
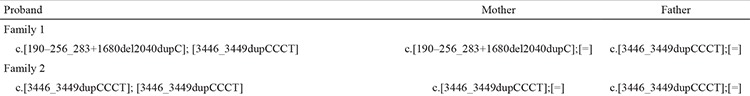
Genotypes of the probands and their parents in *FANCA* gene (NM_000135.2)
